# The Neuroprotection of Liraglutide Against Ischaemia-induced Apoptosis through the Activation of the PI3K/AKT and MAPK Pathways

**DOI:** 10.1038/srep26859

**Published:** 2016-05-31

**Authors:** Huili Zhu, Yusheng Zhang, Zhongshan Shi, Dan Lu, Tingting Li, Yan Ding, Yiwen Ruan, Anding Xu

**Affiliations:** 1 Stroke Center and Department of Neurology, the First Affiliated Hospital, Jinan University, Guangzhou, China; 2 Guangdong Hongkong and Macau Institute of CNS Regeneration (GHMICR), Jinan University, Guangzhou, China; 3 Department of Human Anatomy, Jinan University School of Medicine, Guangzhou, China

## Abstract

Glucagon-like peptide-1 (GLP-1) is an incretin hormone that increases glucose-dependent insulin secretion to reduce the glucose level. Liraglutide, a long-acting GLP-1 analogue, has been found to have neuroprotective action in various experimental models. However, the protective mechanisms of liraglutide in ischaemic stroke remain unclear. Here, we demonstrated that liraglutide significantly decreased the infarct volume, improved neurologic deficits, and lowered stress-related hyperglycaemia without causing hypoglycaemia in a rat model of middle cerebral artery occlusion (MCAO). Liraglutide inhibited cell apoptosis by reducing excessive reactive oxygen species (ROS) and improving the function of mitochondria in neurons under oxygen glucose deprivation (OGD) *in vitro* and MCAO *in vivo*. Liraglutide up-regulated the phosphorylation of protein kinase B (AKT) and extracellular signal-regulated kinases (ERK) and inhibited the phosphorylation of c-jun-NH2-terminal kinase (JNK) and p38. Moreover, the phosphatidylinositol 3-kinase (PI3K) inhibitor LY294002 and/or the ERK inhibitor U0126 counteracted the protective effect of liraglutide. Taken together, these results suggest that liraglutide exerts neuroprotective action against ischaemia-induced apoptosis through the reduction of ROS and the activation of the PI3K/AKT and mitogen-activated protein kinase (MAPK) pathways. Therefore, liraglutide has therapeutic potential for patients with ischaemic stroke, especially those with Type 2 diabetes mellitus or stress hyperglycaemia.

Stroke is the second leading cause of long-term disability and death worldwide^1^. Type 2 diabetes mellitus (T2DM) is one of the major risk factor for ischemia stroke^2^. Therefore, some anti-diabetic drugs may have potential protective effects in stroke, especially in patients with T2DM or hyperglycaemia. Glucagon-like peptide-1 (GLP-1) is secreted from small intestinal L cells in response to food ingestion^3^. GLP-1 and its analogues can stimulate glucose-dependent insulin secretion and insulin gene expression in pancreatic β-cells without causing hypoglycaemia^4^,^5^. Furthermore, GLP-1 and its analogues enable the induction of pancreatic β-cells proliferation and inhibit β-cells apoptosis^6^. Therefore, GLP-1 analogues are approved for clinical use in T2DM. Glucagon-like peptide-1 receptor (GLP-1R) is expressed not only in the pancreas but also in other major mammalian organs, including the lung, kidney, atrial cardiomyocytes, lymphocytes, and the brain, which implies an extensive range of extrapancreatic functions^7^. In the past decade, GLP-1 and its analogues have been reported to exert neurotrophic and neuroprotective actions in neurodegeneration and neurogenesis^8^,^9^.

Liraglutide, one of the long-acting analogues of GLP-1, is used for the treatment of T2DM and is able to move across the blood-brain barrier^10^. Recently, studies have reported that liraglutide exhibited neuroprotection against the damage of cerebral ischaemia^11^,^12^. For example, Gulati *et al.* performed animal studies with the intraperitoneal injection of 50 μg/kg per day liraglutide for 14 days before middle cerebral artery occlusion (MCAO) surgery. Yasuhara *et al.* used a single dose of 700 μg /kg liraglutide 1 hour after reperfusion. These results confirmed the neuroprotective activities of liraglutide. However, the underlying mechanisms accounting for the protective effects of liraglutide on cortical neurons after ischaemia remain unclear, and the methods of treatment need improvement.

In this study, we explored the effect of liraglutide on the oxidative stress and apoptosis of ischaemia-injured neurons and probed its mechanism using primary neurons under oxygen glucose deprivation (OGD) and a rat MCAO model. We demonstrated that liraglutide exhibited protective effects on hypoxic neurons by activating the phosphatidylinositol 3-kinase (PI3K)/protein kinase B (AKT) and mitogen-activated protein kinase (MAPK) pathways and decreasing reactive oxygen species (ROS) *in vitro* and *in vivo*. Furthermore, liraglutide reduced the infarct volume and improved the neurologic deficits of motor and somatosensory function by blocking neuronal apoptosis *in vivo*. Therefore, liraglutide is a useful neuroprotective agent and may have potential therapeutic applications in ischaemic stroke, especially in patients with T2DM or stress hyperglycaemia.

## Results

### Liraglutide reduces infarct volume and improves neurologic functional outcomes in a rat MCAO model

**Figure 1:**
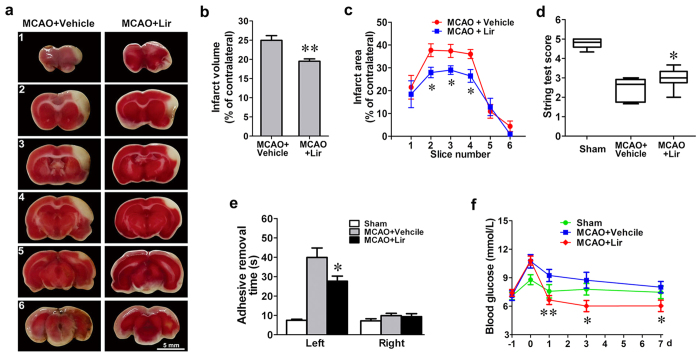
Liraglutide reduces infarct volume and improves neurological deficits. (**a)** Representative photographs of TTC-stained brain slices (2 mm) showing the infarct area 3 days after MCAO. (**b**) Quantitative analysis of the total infarct volume of rats treated with liraglutide (Lir) (n = 6) or vehicle (n = 6). (**c**) Quantitative analysis of the infarct area in each slice from rats treated with Lir or vehicle. (**d**,**e**) Quantitative analysis of the scores of the string test and adhesive removal test of rats treated with Lir (n = 8) or vehicle (n = 8) 7 days after MCAO. (**f**) The changes in the rats’ blood glucose levels. *p < 0.05 and **p < 0.01 *vs*. the MCAO + vehicle group.

To evaluate the protective effect of liraglutide *in vivo*, a dose of liraglutide (100 μg/kg/d) or vehicle was subcutaneously injected into MCAO rats once per day for 1, 3 and 7 days. The infarct area of the cortex was visualized by 2,3,5-Triphenyltetrazolium chloride (TTC) staining 3 days after MCAO surgery. The area of infarction is shown in white in the right cerebral cortex (Fig. 1a). Quantitative analysis showed that the percentage of the infarct volume measured against the contralateral hemisphere was reduced from 25.0 ± 1.2% in the vehicle group to 19.5 ± 0.6% in the liraglutide group (Fig. 1b). Correspondingly, liraglutide significantly reduced the infarct areas in slice 2, 3 and 4 compared to the vehicle group (Fig. 1c). Behavioural assessment showed that String test score was increased from 2.4 in the vehicle group to 3.0 in the liraglutide group, which indicated improved motor function (Fig. 1d). Furthermore, the adhesive removal time of the left forelimb was reduced from 39.9 s in the vehicle group to 27.6 s in the liraglutide group, indicating the relief of somatosensory damage (Fig. 1e). The blood glucose increased from 7.2 ± 0.2 mmol/L to 10.1 ± 0.4 mmol/L after the administration of MCAO alone. In the presence of liraglutide, however, stress-induced hyperglycaemia was improved to 6.7 ± 0.5 mmol/L after 1 day without causing hypoglycaemia (Fig. 1f).

### Liraglutide decreases the death of cortical neurons induced by OGD and ischaemic damage

**Figure 2:**
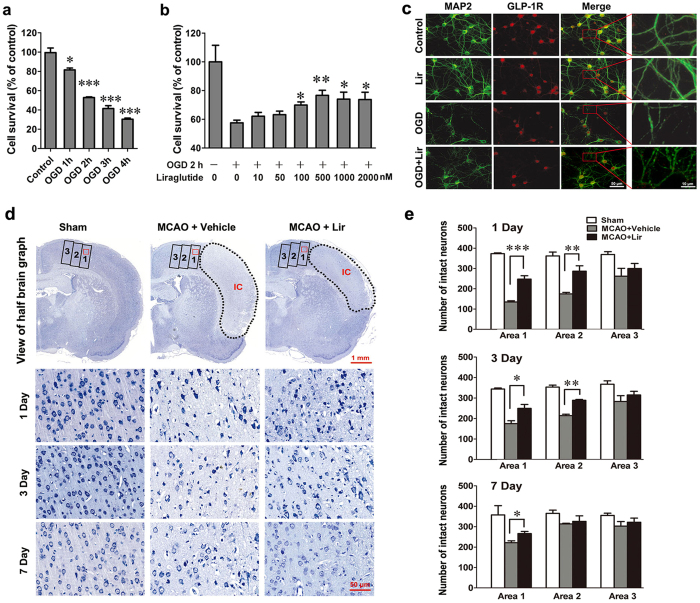
Liraglutide decreases the death of cortical neurons. (**a**) The survival of neurons exposed to OGD for different periods of time. Survival was determined by the CCK-8 kit according to the manufacturer’s protocol (n = 3 experiments, *p < 0.05 and ***p < 0.001 *vs.* control). (**b**) The neurons treated with OGD for 2 h were incubated with Lir at concentrations ranging from 10 to 2000 nM. (n = 3 experiments, *p <  0.05 and **p < 0.01 *vs.* OGD group). (**c**) Immunofluorescence staining of neuronal cultures with anti-MAP2 and GLP-1R antibodies after treatment with OGD or Lir. (**d**) Representative photomicrographs of Nissl-stained brain sections (10 μm). Rats were injected with Lir (n = 12) or vehicle (n = 12) for 1, 3, and 7 days after MCAO. The infarct core (IC) was denoted with a dashed outline and the areas adjacent to the infarct core were marked with rectangles; the rectangular area is 500 × 1200 μm. (**e**) Quantitative analysis showed changes in the number of intact neurons in the area adjacent to the infarction area 1, 3, and 7 days after MCAO. *p < 0.05, **p < 0.01 and ***p < 0.001 *vs.* MCAO+ vehicle group.

First, we explored whether liraglutide could be a protective agent to rescue OGD-caused ischaemic injury *in vitro*. OGD-induced neuronal death occurred in a time-dependent manner, as evidenced by a significant decline in survival at 81.5% for 1 h, 52.8% for 2 h, 41.5% for 3 h and 30.4% for 4 h, respectively (Fig. 2a). We next tested the protective effect of liraglutide on cortical neurons 2 h after OGD damage. The results showed that liraglutide treatment remarkably increased the survival of OGD-treated neurons in a dose-dependent manner, and the concentration of 500 nM had the best effect (Fig. 2b). Microtubule-associated protein 2 (MAP2) is a neuron-specific cytoskeletal protein. Immunofluorescent staining demonstrated that all MAP2-positive cells co-expressed the GLP-1R protein. While OGD caused a loss of dendrite branches in cortical neurons, liraglutide (500 nM) significantly decreased the damage of dendrites and axons caused by OGD (Fig. 2c).

In stroke, the neurons in the core infarct area become irreversibly damaged; therefore, the protection of neurons in the penumbra area is the goal of protective agents. To further investigate the neuroprotective effects of liraglutide in the rat MCAO model, the number of intact neurons was counted in the area adjacent to the infarct core using Nissl staining. As depicted in Fig. 2d, the infarct core (indicated by the dashed outline) and the adjacent area were divided into 3 rectangular areas (1, 2 and 3) according to the distance to the infarct core; the rectangular area is 500 × 1200 μm. The number of intact neurons in the region adjacent to the infarct core was notably decreased and exhibited irregular morphology and disordered arrangement after MCAO (seen in the magnified zone with the red rectangle in Area 1). However, liraglutide treatment significantly improved the number of intact neurons and the morphology and arrangement of cells. To confirm these results, we quantified the number of intact neurons in Areas 1, 2 and 3, respectively. As shown in Fig. 2e, in the liraglutide group, the numbers of intact neurons in Area 1 were 248, 250 and 266 at the 1^st^, 3^rd^ and 7^th^ days after MCAO, respectively. These values were 1.8-, 1.4- and 1.2-fold higher, respectively, than those of the vehicle group. The numbers of intact neurons in the liraglutide group were 1.6- and 1.4-fold higher than those of the vehicle group in Area 2 at the 1^st^ and 3^rd^ day after MCAO, respectively.

### Liraglutide exerts a neuroprotective action against ischaemic injury by suppressing cell apoptosis

**Figure 3:**
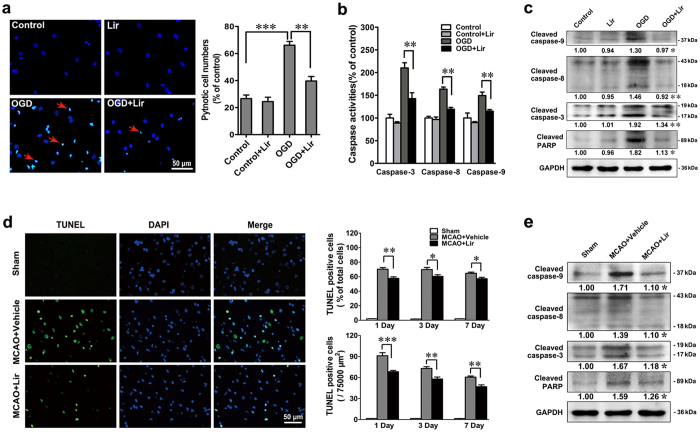
Liraglutide protects neurons by antagonizing the action of apoptosis. (**a**) Hochest33342 staining of neurons after treatment with Lir, OGD or OGD + Lir. The morphology of nuclei was visualized under a fluorescence microscope and the cell numbers were quantified by Image J Software, n = 3 experiments, **p < 0.01 and ***p < 0.001 *vs.* the indicated group. (**b**) The caspase activities of neurons treated with Lir, OGD or OGD + Lir were quantified by fluorogenic substrates of caspase-3, -8 and -9, respectively. (**c**) Western blot analysis for the expression of cleaved caspase-8, -9, and -3 and cleaved PARP *in vitro*. n = 3 experiments, *p < 0.05, **p < 0.01 and ***p < 0.001 *vs.* OGD group. (**d**) TUNEL and DAPI staining of rat brain sections (10 μm) in sham-operated (n = 12), vehicle-treated (n = 12) and Lir-treated (n = 12) groups 1, 3 and 7 days after MCAO. The quantification of TUNEL-positive cells after treatment with Lir or vehicle in the ischaemic region 1, 3 and 7 days after MCAO. (**e**) Western blot analysis of cleaved caspase-9, -8, and -3 and cleaved PARP. *p < 0.05, **p < 0.01 and ***p < 0.01 *vs.* MCAO+ vehicle group.

Liraglutide is capable of inhibiting cellular apoptosis; therefore, we examined the percentage of apoptosis and the expression of proteins related to apoptosis in relation to treatment with liraglutide (500 nM). Hochest33342 staining was performed to visualize the nuclear morphology of the treated neurons. As shown in Fig. 3a, OGD induced nuclear condensation in neurons, as observed by the shrinking and brighter blue dots (denoted by the red arrows). The percentage of pyknotic nuclei dramatically increased to 66.1% after OGD exposure, while liraglutide treatment remarkably decreased the percentage to 39.6%, indicating that liraglutide reversed the apoptosis caused by OGD. The determination of caspase activity by fluorescence probing revealed that liraglutide significantly reduced the OGD-induced activation of caspase-3 (executive caspase), caspase-8 (initiator caspase, Fas/TNF-mediated caspase) and caspase-9 (mitochondrial-mediated caspase) (Fig. 3b). Moreover, the activation of caspases was confirmed by western blot. As shown in Fig. 3c, OGD significantly up-regulated the expression of the cleaved form of caspase-3, -8, and -9 and Poly ADP-ribose Polymerase (PARP). However, this trend was reversed by liraglutide, as shown by the alleviated expression of these proteins.

To confirm whether the neuroprotection of liraglutide was mediated by an anti-apoptosis pathway *in vivo*, terminal-deoxynucleotidyl transferase-mediated nick end labelling (TUNEL) staining was employed to detect the deoxyribonucleic acid (DNA) damage caused by MCAO in the cortex. As shown in Fig. 3d, there was no detectable fluorescence of nuclei in the sham group initially, but strong positive green fluorescent nuclei were observed in the vehicle group 24 h after MCAO. Liraglutide successfully alleviated the DNA damage caused by MCAO, as evidenced by the decreased intensity of TUNEL fluorescence. The quantitative data showed that the percentage of TUNEL-positive cells (out of the total cell number) and the absolute number of TUNEL-positive cells were both deceased in the liraglutide group compared to the vehicle group 1, 3 and 7 days after MCAO. Western blot analysis showed that ischaemia alone increased neuronal apoptosis, as reflected by the up-regulation of cleaved caspase-3, -8,-9 and PARP. Additionally, liraglutide significantly down-regulated these proteins compared to the vehicle group (Fig. 3e). These results confirmed the anti-apoptotic effects of liraglutide in ischaemic stroke.

### Liraglutide decreases the generation of ROS in hypoxic neurons

**Figure 4:**
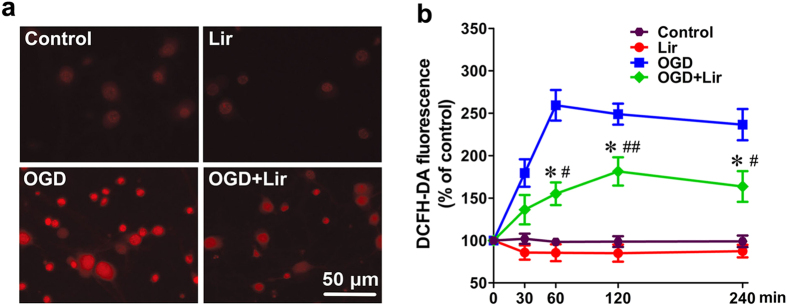
Liraglutide reduces ROS generation. (**a**) Representative fluorescence images showed changes in intracellular ROS levels as measured by the DCFH-DA fluorescence intensity of neurons treated with or without 500 nM Lir after exposure to OGD. (**b**) The primary neurons incubated with 10 μM DCFH-DA were exposed to Lir, OGD or OGD + Lir for 30, 60, 120 and 240 min, respectively. The intracellular ROS level was measured by the fluorescence intensity of DCFH-DA (n = 3 experiments, *p < 0.05 *vs.* OGD group, ^#^*p* < 0.05 and ^##^*p* < 0.01 *vs.* control group).

Numerous studies have reported that ischaemia triggers the overproduction of ROS and subsequently causes DNA damage and apoptosis^13^. In this study, the intracellular ROS level was quantified using dichlorofluorescein diacetate (DCFH-DA) as the fluorescence probe to visualize and quantify the intracellular production of ROS. As illustrated by Fig. 4a, OGD induced the overproduction of ROS in cortical neurons, as visualized by strong red fluorescent staining. However, liraglutide (500 nM) treatment significantly reduced the intensity of red fluorescence caused by OGD. Quantitative analysis showed that the rates of ROS accumulation in the OGD group markedly increased to the peak value of 252.7% at 60 min, then gradually decreased to 248.9% at 120 min and 236.5% at 240 min compared to the control group. On the other hand, liraglutide exhibited ROS levels of 155.1% at 60 min, 181.4% at 120 min and 163.7% at 240 min compared to the control group (Fig. 4b). These results suggested that liraglutide is able to inhibit the generation or increase the scavenging of ROS in hypoxic neurons.

### Mitochondria are involved in the anti-apoptotic effect of liraglutide

**Figure 5:**
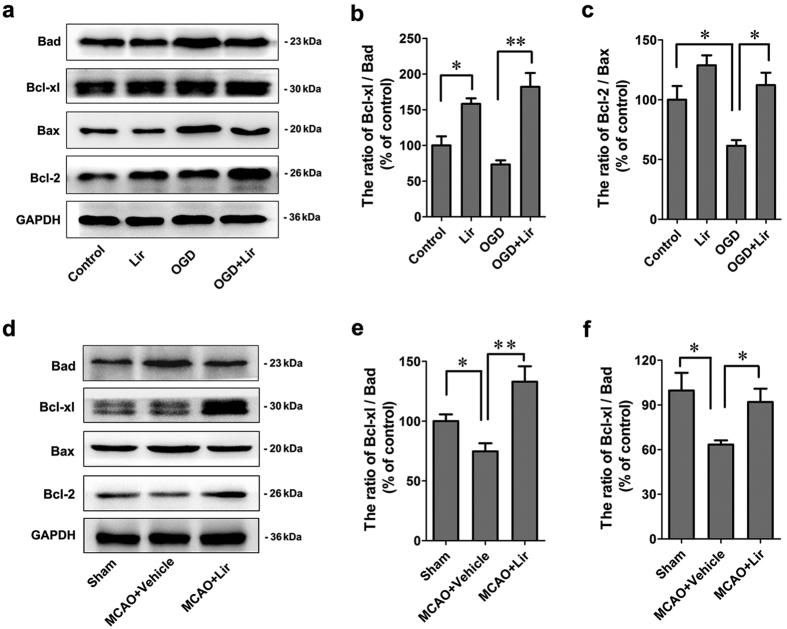
Bcl-2 family proteins are involved in the anti-apoptosis effect of liraglutide. (**a**) Western blot analysis of the expression of Bad, Bcl-xL, Bax and Bcl-2 *in vitro*. (**b**,**c**) The expression ratio of Bcl-xL/Bad and Bcl-2/Bax was quantified by Quantity One and is represented as a histogram. (**d**) Western blot analysis for the expression of Bad, Bcl-xL, Bax and Bcl-2 *in vivo*. (**e**,**f**) The expression ratio of Bcl-xL/Bad and Bcl-2/Bax was quantified by Quantity One and is represented as a histogram. n = 3 experiments, *p < 0.05 and **p < 0.01 *vs.* the indicated group.

The Bcl-2 family of proteins, the essential apoptotic regulators of mitochondria, were investigated in primary neurons after OGD or liraglutide treatment by western blot. As shown in Fig. 5a, liraglutide increased the expression level of the anti-apoptotic Bcl-2 and Bcl-xl proteins, while it decreased the expression level of the pro-apoptotic Bax and Bad proteins. The corresponding quantitative assessment showed that liraglutide increased the ratio of Bcl-xl/Bad and Bcl-2/Bax relative to the OGD group (Fig. 5b,c).

Similarly, liraglutide also increased the expression level of the anti-apoptosis proteins Bcl-2 and Bcl-xl, while it decreased the expression of the pro-apoptotic Bax and Bad proteins in MCAO rats (Fig. 5d). The ratio of Bcl-xl/Bad and Bcl-2/Bax was markedly increased in the liraglutide group relative to the OGD group (Fig. 5e,f), which suggested that liraglutide attenuated the apoptosis signalling pathway by regulating mitochondrial function in hypoxic and ischaemic neurons.

### Liraglutide protects cortical neurons from apoptosis by activating the PI3K/AKT and MAPK pathways

**Figure 6:**
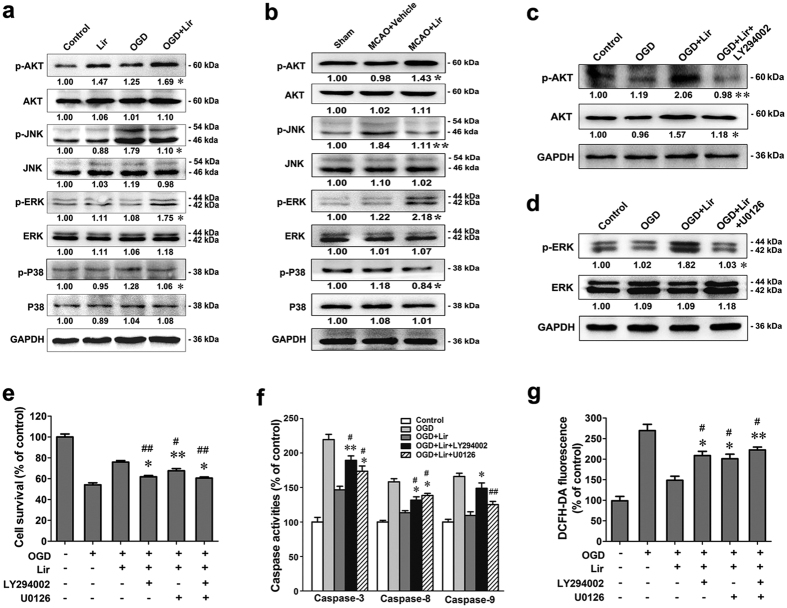
Liraglutide protects neurons by activating the PI3K/AKT and MAPK pathways. (**a**) Western blot analysis of the expression of AKT, p-AKT, ERK, p-ERK, p38, p-p38, JNK and p-JNK in primary neurons (n = 3 experiments, *p < 0.05 *vs*. OGD group). n = 3 experiments, MCAO + vehicle group). (**c**) Expression of AKT and p-AKT in neurons after treatment with Lir and the PI3K inhibitor LY294002 *in vitro* (n = 3 experiments, **p < 0.01 *vs*. OGD + Lir group). (**d**) Expression of ERK and p-ERK after treatment with the Lir and ERK inhibitor U0126 *in vitro* (n = 3 experiments, *p < 0.05 *vs*. OGD + Lir group). (**e**) Cell viability was measured by the CCK-8 method following treatment with LY294002 and/or U0126. (**f**) The caspase activity of neurons treated with LY294002 and U0126 *in vitro*. (**g**) The intracellular ROS level measured by the DCFH-DA fluorescence intensity of neurons treated with LY294002 and/or U0126 after exposure to OGD for 60 min (n = 3 experiments, *p < 0.05 and **p < 0.01 *vs*. OGD + Lir group, ^#^*p* < 0.05 and ^##^*p* < 0.01 *vs*. OGD group).

Several kinases, such as PI3K/AKT and the MAPK family, play an important role in the GLP-1R mediated anti-apoptosis signalling pathways in many types of cells^14^,^15^,^16^. To identify whether these kinases are also involved in liraglutide-induced neuroprotection, western blot analysis was performed. The results showed that liraglutide increased the expression of phosphorylated AKT and ERK but decreased the expression of phosphorylated p38 and c-jun-NH2-terminal kinase (JNK) *in vitro* (Fig. 6a). Animal studies also showed that liraglutide increased the expression of phosphorylated AKT and ERK but decreased the expression of phosphorylated p38 and JNK in the right cerebral cortex of MCAO rats (Fig. 6b).

To further confirm the role of AKT and ERK in liraglutide-induced neuroprotection, neurons were treated with inhibitors of PI3K (LY294002) and/or ERK (U0126), respectively. The expression levels of phosphorylated AKT and ERK in neurons were remarkably suppressed by LY294002 and U0126, respectively, compared to the OGD plus liraglutide group (Fig. 6c,d). Although liraglutide increased the survival rate of neurons to 76%, treatment with either LY294002 or U0126 decreased liraglutide-mediated survival to 62% and 68%, respectively. Furthermore, co-treatment with LY294002 and U0126 decreased the survival to 60% (Fig. 6e). Consistent with these results, treatment with LY294002 significantly increased the intracellular levels of caspase-3, -8, and -9, while treatment with U0126 increased the intracellular level of caspase-3 and -8 compared to the OGD plus liraglutide group (Fig. 6f).

**Figure 7:**
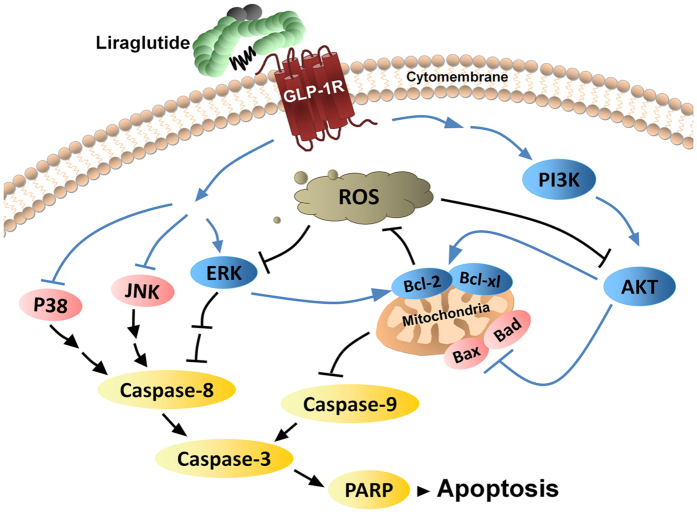
Schematic representation of the proposed anti-apoptotic signalling pathways triggered by liraglutide in ischaemic neurons. Liraglutide activates the PI3K/AKT and MAPK pathways, and phosphorylated Akt and ERK up-regulate the expression level of Bcl-2 and Bcl-xl. Bcl-2 and Bcl-xl can inhibit ROS generation, and the reduced level of ROS in turn decreases the inhibition of AKT and ERK activity, which ultimately inhibits the extrinsic and intrinsic apoptotic signalling pathways to block apoptosis. The pathways supported by our current data are indicated by blue arrows and the pathways that were shown in previous studies are indicated by black arrows.

We also examined the intracellular ROS level in neurons treated with LY294002 and/or U0126 after exposure to OGD for 60 min. As shown in Fig. 6g, treatment with either LY294002 or U0126 increased intracellular ROS accumulation compared to the OGD plus liraglutide group. Interestingly, the two inhibitors together did not completely block the liraglutide’s suppression of OGD-induced ROS in ischaemic neurons. These results indicated that liraglutide decreased the generation of ROS in hypoxic neurons in part by the PI3K/AKT and MAPK pathways. Based on the obtained results, the mechanisms of action and the underlying signalling pathways of liraglutide were proposed in Fig. 7.

## Discussion

Neurons are the most oxygen-sensitive cells in the human body. Hypoxia in neurons can cause lipid peroxidation, mitochondrial dysfunction, ROS overproduction and the obstruction of ATP synthesis, which all ultimately result in cell apoptosis. Numerous studies have shown that the analogues of GLP-1 can antagonize the apoptosis of pancreatic β-cells due to their anti-oxidative activity and their activation of anti-apoptosis pathways^17^. Therefore, it is possible that liraglutide, an analogue of GLP-1, may also inhibit the overproduction of ROS that occurs in ischaemic neurons to decrease the incidence of apoptosis in cerebral ischaemia. In this study, we report for the first time that liraglutide can effectively antagonize the neuronal damage caused by hypoxia and hypoglycaemia via the activation of anti-apoptosis pathways *in vitro*. Moreover, liraglutide reduced the infarct volume and notably improved the neurologic deficits of motor and somatosensory function *in vivo*. Mechanistic studies additionally revealed that liraglutide triggered the PI3K/AKT and MAPK pathways and alleviated ROS levels in ischaemic neurons. These results may enhance our understanding of the neuroprotection of liraglutide against ischaemic stroke and provide useful information for the future treatment of this disease.

Recent research showed that GLP-1R has the promising ability to generate neuroprotection against neurological disorders, including Alzheimer’s disease (AD), Parkinson’s disease (PD), amyotrophic lateral sclerosis (ALS) and ischaemic stroke^18^,^19^. Although many new drugs have been demonstrated to have a significant neuroprotective ability based on laboratory results, these candidate drugs have not been approved by clinical trials. However, edaravone, a free radical scavenger, has been demonstrated to show efficient neuroprotective effects of ischaemic stroke in some clinical trials^20^,^21^. These results suggest that free radicals play an important role in the aetiology of cerebral ischaemia. Cells undergoing ischaemic damage produce excess amounts of ROS, which potentiates lipid peroxidation and mitochondrial dysfunction. Subsequently, cytochrome C is released from mitochondria, which could bind to cleaved caspase-9 to form apoptotic bodies and trigger intrinsic apoptotic pathways. Additionally, ROS stimulates the cleavage of caspase-8 via multiple pathways and triggers extrinsic apoptotic signalling pathways for the inhibition of cell survival^22^. In this study, we confirmed that liraglutide has the ability to inhibit the generation or increase the scavenging of ROS in hypoxic neurons.

Many studies have shown that the activation of PI3K/AKT and ERK can effectively inhibit ROS generation by regulating the expression of the Bcl-2 family^23^. As important anti-oxidative proteins, Bcl-2 and Bcl-xl efficiently scavenge free radicals and inhibit the formation of superoxide, which consequently alleviates the oxidative damage caused by ROS overexpression in ischaemic neurons^24^. Meanwhile, Bcl-2 has strong antioxidant activity, and PI3K/AKT and ERK can up-regulate the expression level of Bcl-2 to inhibit ROS generation in ischaemic damage^25^,^26^,^27^. Interestingly, ROS inhibits AKT and ERK phosphorylation and promotes cell apoptosis in turn^28^,^29^. The PI3K/AKT pathway is central to the anti-apoptotic actions of GLP-1 in pancreatic β-cells. The elevated level of phosphorylated AKT induced by GLP-1 activates downstream proteins, including nuclear factor-κB (NF-κB)^30^ and pancreatic and duodenal homeobox 1 (PDX-1)^31^, while reducing JNK and glycogen synthase kinase-3β (GSK-3β) activity in the pancreas^32^. Moreover, increasing numbers of studies have reported that the analogues of GLP-1 enable the inhibition of the apoptosis of neogenic rat myocardial cells by triggering AKT and ERK signalling under hypoxic environments^33^. Likewise, the parallel biochemical cascades triggered by GLP-1 and its analogues may contribute to the protection of ischaemic neurons in a similar fashion. In this study, we found that liraglutide activated AKT and ERK in ischaemic neurons, which subsequently up-regulated the expression of Bcl-2 and Bcl-xl and decreased intracellular ROS generation. The reduced level of ROS in ischaemic neurons reduced the inhibition of AKT and ERK activity in turn. Therefore, liraglutide could promote the expression of pro-survival proteins and simultaneously inhibit pro-apoptotic proteins by triggering PI3K/AKT and MAPK-mediated signalling and promoting the overall anti-apoptotic effects of ischaemic neurons (Fig. 7). Interestingly, a combination of PI3K and ERK inhibitors did not completely eliminate the inhibitory effect of liraglutide on ROS, indicating the involvement of additional signalling pathways.

Clinically, the recommended dose of liraglutide for T2DM in adults ranges 0.6 to1.8 mg/d, which corresponds to approximately 50 to 150 μg/kg/d using a pharmacokinetic formula for humans and rats. Yasuhara *et al.* previously showed that the intraperitoneal injection of a single dose (700 μg/kg) of liraglutide one hour after reperfusion reduced the infarct volume by 17.6% 24 h after MCAO^11^. However, the dosage of liraglutide exceeded its recommended dosage for clinical use and may increase the risk of hypoglycaemia in patients with stroke. Gulati’s group reported that intraperitoneal injection with liraglutide (50 μg/kg/d) for 14 days before MCAO reduced infarct volume by 13.8% 24 h after MCAO surgery^12^. Because the timing of the stroke is unpredictable, the early use of liraglutide before MCAO is not consistent with the progress of treatment. In this study, the MCAO model was established and 100 μg/kg/d liraglutide was subcutaneously injected into the rats 1 hour after surgery for 1, 3 and 7 days, which simulated clinical situations. We observed a decreased infarct volume of 21.9% in the liraglutide group 3 days after MCAO. These results indicate that liraglutide can protect neurons against ischaemic injury under both conditions, before or after acute stage ischaemic stroke. However, a moderate dose of liraglutide administered continuously over a period of time after acute ischaemic stroke may achieve improved neurological outcomes and is easy to implement. This study provides experimental evidence for the efficient clinical use of liraglutide.

It should be noted that the anti-apoptotic action of liraglutide may not be the sole factor that accounts for liraglutide-mediated neuroprotection. Numerous reports have suggested that the analogues of GLP-1 may also promote the proliferation of vascular endothelial cells and thus provoke the growth of new vessels in cardiac ischaemic models^34^,^35^. Other studies have focused on the efficacy of GLP-1R agonists’ regulation of the inflammatory reaction, which occurs in T2DM and ischaemic diseases^36^. Therefore, pro-angiogenic and anti-inflammatory mechanisms may also play a role in the neuroprotection of liraglutide after ischaemia. In this paper, we only reported the neuroprotective effect of liraglutide on ischaemic neurons and its underlying mechanism. Future studies should examine the impact of liraglutide on the viability of vascular endothelial cells and its effect on inflammatory mechanisms in focal cerebral ischaemia.

Overall, we concluded that liraglutide can activate the pro-survival proteins AKT and ERK and inhibit the anti-survival proteins p38 and JNK, which subsequently inhibit the extrinsic and intrinsic apoptotic signalling pathways to block apoptosis. Furthermore, liraglutide inhibits intracellular ROS generation and improves mitochondrial function by the PI3K/AKT and MAPK signalling pathways in ischaemic neurons. Liraglutide therefore reduced the infarct volume and improved the neurologic deficits in motor and somatosensory function without producing hypoglycaemia in a rat MCAO model. Collectively, this study provides preclinical evidence for the use of liraglutide in the treatment of ischaemic stroke, especially in patients with T2DM or stress hyperglycaemia.

## Materials and Methods

### Isolation and culture of primary neurons

Cerebral cortical neurons were dissociated from neonatal Sprague-Dawley rats as previously described with slight modifications^37^. Briefly, neonatal rats were anaesthetized with 2% isoflurane and the cortex was dissected and digested with 0.125% trypsin for 30 min at 37 °C. Neurons were plated on 0.0125% poly-L-lysine-coated culture dishes in Neurobasal medium (Gibco, USA) with B27 (Gibco, USA) in a humidified atmosphere of 5% CO_2_ at 37 °C. After 10 days, neurons were placed into an anaerobic chamber (Coy Laboratories, USA) and the culture medium was replaced with a balanced salt solution (116 mM NaCl, 5.4 mM KCl, 1 mM NaH_2_PO_4_, 1.8 mM CaCl_2_, 26.2 mM NaHCO_3_, 5 mM HEPES, pH 7.4) aerated with nitrogen to remove the oxygen. Some cultures were treated with liraglutide peptide (500 nM, PeproTech, USA) and/or LY294002 (10 nM, Sigma, USA) or U0126 (10 nM, Sigma, USA) during the OGD insult. At the end of the insult (2 h), the OGD medium was replaced with the original medium and then incubated for 24 h in a humidified atmosphere of 5% CO_2_ at 37 °C for further experiments. The animal experimental protocols were approved by the Animal Care and Use Committee of Jinan University. All experimental protocols involving rats were approved by the ethical committee of Jinan University and performed in accordance with approved guidelines and regulations.

### Cell survival assays

Cell survival was determined using the cell counting kit-8 (CCK-8) (Dojindo, Japan) according to the manufacturer’s instructions. Briefly, the treated neurons were loaded into 96-well plates and replenished with 100 μl fresh DMEM medium (Gibco, USA) with 10 μl CCK-8 solution added to each well. After incubation at 37 °C for 2 h, cell survival was determined by optical density values read at 450 nm.

### Immunofluorescence assays

Primary neurons were fixed in 4% paraformaldehyde for 30 min at room temperature. After incubation in 1% BSA, 5% goat serum and 0.2% Triton X-100 in PBS for 2 h at 4 °C, GLP-1R (Abcom, UK) and MAP2 antibodies (Millipore, USA) from different species were sequentially applied overnight at 4 °C and corresponding secondary antibodies were applied for 1 h at room temperature, followed by counterstaining with 4,6-diamidino-2-phenylindole dihydrochloride (DAPI).

### Measurement of ROS generation

Neurons were washed twice with PBS and incubated with DCFH-DA (Sigma, USA) to a final concentration of 10 μM at 37 °C for 30 min. Intracellular ROS generation was monitored by measuring the fluorescence intensity of cells with a microplate reader (PerkinElmer, USA), with excitation and emission wavelengths set at 500 nm and 529 nm, respectively.

### Caspase activity assay

Sample proteins (100 μg/well) were placed in 96-well plates and Ac-DEVD-AMC, Ac-IETD-AMC and Ac-LEHD-AMC (Millipore, USA) were added to detect caspase-3, -8 and -9, respectively. After incubation at 37 °C for 2 h, the caspases’ activities were determined by fluorescence intensity with the excitation and emission wavelengths set at 380 and 440 nm, respectively.

### Western blot analysis

The expression of proteins extracted from the primary neurons and the right cerebral cortex of rats was analysed by Western blot. The protein samples were separated on a 12% sodium dodecyl sulfate (SDS)/polyacrylamide gel and transferred onto polyvinylidene fluoride membranes (Millipore, USA). The membranes were incubated with GLP-1R (Abcom, UK), phosphorylated AKT, ERK, p38, JNK and total AKT, ERK, p38, JNK, Bcl-2, Bax, Bcl-xl, Bad, cleaved caspase-3, -8, and -9, cleaved PRAP and GAPDH antibodies (Cell Signaling Technology, USA) at 4 °C overnight. Immunoreactive bands were visualized by increased chemiluminescence (Millipore, USA) using corresponding horseradish peroxidase-conjugated IgG secondary antibodies (Cell Signaling Technology, USA). The images were captured by the gel imager (UVITEC, UK) and quantified using Quantity One software (Bio-Rad, USA).

### Animals and MCAO model

A total of 84 male Sprague Dawley rats (300–350 g) were purchased from the Animal Experiment Center of Southern Medical University, Guangzhou, China. Sixty rats were subjected to MCAO and 24 rats served as sham-operated controls. The MCAO model was performed as previously described^38^. Briefly, the rats were anaesthetized with chloral hydrate (0.3 mg/kg) by intraperitoneal injection. Under an operating microscope, the right middle cerebral artery was exposed through a burr hole and occluded by electrocoagulation. Sham-operated animals underwent the same surgical procedures except for the electrocoagulation of the middle cerebral artery. At 1 hour after MCAO, rats in the liraglutide and vehicle groups were subcutaneously injected with liraglutide (100 μg/kg/d, Novo Nordisk, Denmark) and isopyknic vehicle, respectively, then continuously injected once a day for 1, 3 and 7 days. The animal experimental protocols were approved by the Animal Care and Use Committee of Jinan University. All experimental protocols involving rats were approved by the ethical committee of Jinan University and performed in accordance with approved guidelines and regulations.

### Behavioural tests

All rats were trained for behavioural test for 7 days before MCAO. After 7 days of MCAO, the string test was performed to assess the motor function as previously described^39^. Briefly, a string (2 mm in diameter and 50 cm in length) was stretched tightly between two vertical supports placed at a height of 40 cm above a flat surface. The rat was placed on the string at a point midway between the supports. The rats were rated by the experimenter according to the following system: 0 = falls off, 1 = hangs onto the string by two forepaws, 2 = hangs onto the string by two forepaws but attempts to climb onto the string, 3 = hangs onto the string by two forepaws plus one or both hind paws, 4 = hangs onto the string by all four paws plus the tail wrapped around string, 5 = escape. The adhesive removal test was performed to assess the somatosensory deficit as previously described at 7 days after MCAO^40^. Briefly, rats were placed in a box for 1 min in order to familiarize them to the environment. Then, two small adhesive paper dots (0.4 × 1 cm) were accurately attached to the wrist of each forelimb with equal pressure. The time to remove each stimulus from the forelimbs was recorded in three trials for each forepaw.

### Measurement of blood glucose

Blood samples (200 μl) were collected from the ophthalmic venous plexus 1 day before MCAO, during the process of surgery, and 1, 3, and 7 days after MCAO. Blood glucose was measured by a glucometer (Roche, Germany) according to the manufacturer’s instructions.

### TTC staining

Twelve MCAO rats were randomly divided into liraglutide and vehicle groups. The rats were deeply anaesthetized with chloral hydrate and perfused with cold saline. The brains were quickly removed and chilled at −80 °C for 5 min. Each brain was coronally sliced into six sections (2 mm intervals). The sections were stained with 2% TTC (Sigma, USA) at 37 °C for 30 min, then fixed in 4% formaldehyde overnight at 4 °C. The relative infarct volume was presented as a volume percentage of the lesion compared to the contralateral hemisphere and was quantitatively analysed by Image J software (National Institutes of Health, USA).

### Nissl staining

Thirty-six rats were divided into sham, liraglutide and vehicle groups and anaesthetized and perfused with 100 ml saline and subsequently with 250 ml 4% paraformaldehyde 1, 3 and 7 days after MCAO. Brains were removed and placed into 4% paraformaldehyde for 3 days at 4 °C. Then, the brain tissues were embedded in paraffin and sectioned coronally at 10 μm thicknesses with a paraffin slicer. Sections were dehydrated with ethanol and treated with xylene, followed by incubation with 1% cresyl violet (Sigma, USA) solution for 5 min at 50 °C. They were then mounted after dehydration with ethanol.

### TUNEL staining

The fragmentation of genomic DNA was detected by *in situ* staining of DNA ends with TUNEL according to the manufacturer’s instructions (Roche, Germany). Briefly, a total volume of 100 μl of the terminal deoxy-transferase reaction mixture was incubated with the 36 sections for 1 h at room temperature in the dark. DAPI staining was used to count the total number of nuclei.

### Statistical analysis

All data were analysed with GraphPad PRISM 5.0 (GraphPad software, USA) software using the Student’s t-test or one-way ANOVA. Ranked data were analysed with the Kruskal-Wallis test. All quantitative values were expressed as the mean ± SEM. Differences were deemed significant when *P* < 0.05.

## Additional Information

**How to cite this article**: Zhu, H. *et al.* The Neuroprotection of Liraglutide against Ischaemia-induced Apoptosis through the Activation of the PI3K/AKT and MAPK Pathways. *Sci. Rep.* **6**, 26859; doi: 10.1038/srep26859 (2016).
